# Reduction of Mitoferrin Results in Abnormal Development and Extended Lifespan in *Caenorhabditis elegans*

**DOI:** 10.1371/journal.pone.0029666

**Published:** 2012-01-11

**Authors:** Yaguang Ren, Su Yang, Guoqiang Tan, Wei Ye, Danhui Liu, Xu Qian, Zhongying Ding, Yuhong Zhong, Jingrui Zhang, Dandan Jiang, Yuhong Zhao, Jianxin Lu

**Affiliations:** Key Laboratory of Laboratory Medicine, Ministry of Education of China, Zhejiang Provincial Key Laboratory of Medical Genetics, School of Laboratory Medicine and Life Science, Wenzhou Medical College, Wenzhou, Zhejiang, China; Thomas Jefferson University, United States of America

## Abstract

Iron is essential for organisms. It is mainly utilized in mitochondria for biosynthesis of iron-sulfur clusters, hemes and other cofactors. Mitoferrin 1 and mitoferrin 2, two homologues proteins belonging to the mitochondrial solute carrier family, are required for iron delivery into mitochondria. Mitoferrin 1 is highly expressed in developing erythrocytes which consume a large amount of iron during hemoglobinization. Mitoferrin 2 is ubiquitously expressed, whose functions are less known. Zebrafish with *mitoferrin 1* mutation show profound hypochromic anaemia and erythroid maturation arrests, and yeast with defects in *MRS3/4*, the counterparts of *mitoferrin 1/2*, has low mitochondrial iron levels and grows poorly by iron depletion. Mitoferrin 1 expression is up-regulated in yeast and mouse models of Fiedreich's ataxia disease and in human cell culture models of Parkinson disease, suggesting its involvement in the pathogenesis of diseases with mitochondrial iron accumulation. In this study we found that reduced mitoferrin levels in *C. elegans* by RNAi treatment causes pleiotropic phenotypes such as small body size, reduced fecundity, slow movement and increased sensitivity to paraquat. Despite these abnormities, lifespan was increased by 50% to 80% in N2 wild type strain, and in further studies using the RNAi sensitive strain *eri-1*, more than doubled lifespan was observed. The pathways or mechanisms responsible for the lifespan extension and other phenotypes of *mitoferrin* RNAi worms are worth further study, which may contribute to our understanding of aging mechanisms and the pathogenesis of iron disorder related diseases.

## Introduction

Iron is essential for organisms. It is mainly utilized in mitochondria for biosynthesis of hemes, iron-sulfur clusters (Fe-S clusters) and other cofactors [1]–[3]. Hemes are found in hemoglobin, myoglobin, and cytochromes [3]. Fe-S clusters are cofactors of many proteins involved in respiration, transcription, amino acid synthesis and other processes [1], [4], [5]. However, too much iron is detrimental, and iron accumulation in mitochondria is associated with many human genetic disorders, e.g., Fiedreich's ataxia (FRDA), Sideroblastic anemias (SA), ISCU Myopathy, Alzheimer's Disease (AD), Parkinson's disease (PD) and Multiple Sclerosis (MS) [6]–[12]. Aging is a risk factor for these diseases, most of which occur at a high rate of frequency at old age. Iron accumulation is also accompanied with aging [13], [14]. The content of iron is increased in organs of aged animals and during aging our body accumulates a brown material in the tissues known as lipofuscin (age-pigment), which is a mass of fat and iron [15]–[17].

The mechanisms regulating mitochondrial iron homeostasis are largely unknown. Iron delivery into mitochondria is facilitated by mitoferrin 1 and mitoferrin 2, two homologous proteins of the mitochondrial solute carrier family [18], [19]. Down-regulation of mitoferrin 1 and/or mitoferrin 2 levels by RNAi treatment result in decreased mitochondrial iron content and reduced iron-sulfur cluster and heme synthesis [19]. Mitoferrin 1 is highly expressed in haematopoietic tissues and is essential for erythroid iron assimilation. Zebrafish with *mitoferrin 1* mutation shows profound hypochromic anaemia and erythroid maturation arrests [19], [20]. Targeted deletion of mouse *mitoferrin 1* leads to anemia and protoporphyria [21]. Ferrochelatase, the terminal heme synthesis enzyme catalyzing the insertion of iron into protoporphyrin IX during heme production, and abcb10, a mitochondrial inner membrane ATP-binding cassette transporter induced during erythropoiesis in hematopoietic tissues, can form complex with mitoferrin 1 during mouse erythroleukemia cell differentiation [22], [23]. Mitoferrin 2 is widely expressed in most tissues and possibly functions to maintain the base line levels of mitochondrial iron in non-erythroid cells [19], [20]. Less is known about mitoferrin 2 due to a lack of gene knockout models. Invertebrates such as *Drosophila melanogaster*, *Caenorhabditis elegans*, sea urchin, bee, wasp, mosquito, and flour beetle have a single mitoferrin protein which shows similarity to vertebrate mitoferrin 2 due to lack of erythropoiesis [24], [25]. Mitoferrin is essential for male fertility of Drosophila [25]. Yeast with defects in *MRS3/4*, counterparts of *mitoferrin 1/2*, grows poorly under low iron conditions [26]–[28]. Cross-species gene complement experiments showed that *mitoferrin* genes are conserved in evolution. For example, defects of yeast *MRS3/4* mutant can be rescued by zebrafish *mfrns* and murine *mfrn1* can complement zebrafish mutant [19], [20].

Mitoferrin is also involved in pathogenesis of human genetics diseases. The expression of mitoferrin is up-regulated in yeast and mouse models of Fiedreich's ataxia (FRDA) and in human cell culture models of Parkinson disease [29], [30], which may contribute to mitochondrial iron accumulation and cytosolic iron deficiency, primary symptoms of those diseases.

The nematode *Caenorhabditis elegans* is an animal model widely used for studies of subjects including development, cell death, aging and diseases [31]–[33]. It is easy to maintain in laboratory, it is transparent and has a lifecycle of only 3 days at 20°C. Many experimental technologies such as RNA interference (RNAi) and genetic analysis are extensively used in *C. elegans* [34], [35]. In this study we found that *C. elegans* feeding on E.coli HT115 (DE3) strain containing RNAi construct targeting *W02B12.9* (worm *mitoferrin*) showed pleiotropic phenotypes such as small body size, reduced fecundity, slowed movement and increased sensitivity to paraquat. Despite these abnormities, lifespan was increased by 50% to 80% in N2 wild type strain, and in further studies using the RNAi sensitive strain *eri-1*, more than doubled lifespan was observed. Although *mfn-1* RNAi worms were smaller and produced fewer progenies than control, their growth and reproduction periods seem normal, neither prolonged nor shortened. Further investigation into the pathways or mechanisms responsible for the increased longevity and other phenotypes may contribute to our understanding of aging and may have implications in treatment of diseases associated with mitochondrial iron accumulation.

## Materials and Methods

### Strains and worm culture

The *C. elegans* strains used in the experiments are wild type Bristol N2 and RNAi hypersensitive strain GR1373 *eri-1(mg366) IV*, supplied by the Caenorhabditis Genetics Center (University of Minnesota, MN). Worms were cultured and maintained on NGM plates as described [36]. Unless stated otherwise, worms were cultured at 20°C.

### RNAi vector construction

Fragment used for RNAi constructs was obtained by polymerase chain reaction (PCR) from *C. elegans* genomic DNA that was isolated using standard methods [37]. The fragment obtained corresponds to a segment of the second exon of *W02B12.9*. Sequence specificity of the fragment for RNAi was confirmed by blast searches against worm database. Primers used for PCR were:

Forward primer: 5′-GTTAGATCTCTGAAACGAAATGTCCA-3′ (Bgl II enzyme site was underlined);
Reverse primer: 5′-TAAGGTACCAAGTCCTCCCGCAA-3′ (Kpn I enzyme site was underlined).

PCR product was cloned into l4440 vector. Another RNAi vector targeting a different region of *W02B12.9* was constructed by similar method, except that cDNA was used as the template. Primers used for PCR were:

Forward primer: 5′-CAGAGATCTAGGACTTGCCGGCGGATTGG-3′ (Bgl II enzyme site was underlined);
Reverse primer: 5′-CGCAAGCTTTCAACTCGAATGACCTCCT-3′ (Hind III enzyme site was underlined).

E. coli RNase III-deficient strain HT115 (DE3) transformed by the recombinant RNAi vector was selected on agar plates containing 100 µg/ml ampicillin and 12.5 µg/ml tetracyclin. The authenticity of the recombinant RNAi constructs was confirmed by sequencing.

### Bacterially mediated RNAi

Single clone of HT115 (DE3) bacteria transformed by *W02B12.9* RNAi construct was inoculated in LB medium containing 50 µg/ml ampicillin and let grow overnight at 37°C and 250 rpm. Over night culture was diluted 1∶100 in 3 ml fresh LB medium containing 50 µg/ml ampicillin and allowed to grow for 8–10 hours. Then the bacteria was seeded onto NGM plates containing 50 µg/ml ampicillin and 1 mM IPTG and was induced over night at room temperature. L4 staged hermaphrodites were transferred onto the bacterial lawn and let grow at 20°C for RNAi to take effect. Worms feeding on bacteria transformed by empty vector l4440 were used as control. The procedures were essentially described [38].

### Locomotive rate assay

Several L4 staged hermaphrodites were transferred onto *W02B12.9* RNAi or l4440 RNAi plates respectively and let grow for three or four days for RNAi to take effect. One-day old F1 adults (24 hours after L4 stage was defined as one-day old and L4 stage was defined as day 0) were transferred onto fresh plates (without bacteria) for locomotive rate assay. Locomotive activity was determined by counting the number of front body bends in 20 seconds using an inverted microscope. When the anterior body region of the worm just behind the pharynx reached a maximum bend in the opposite direction from the last bend, the body bend count was advanced by one. The body bend values of five animals on each plate were manually counted and averaged in every paired experiment.

### Body length measurement

Several L4 staged hermaphrodites were transferred onto *W02B12.9* or l4440 RNAi plates respectively and let grow for three or four days for RNAi to take effect. Five to seven young adult hermaphrodites of F1 generation were transferred onto fresh prepared RNAi plate and were removed two hours later, about dozens of synchronized eggs should be laid during this period. The F2 generation on the plate were photographed every twenty four hours until growth stopped using a Nikon Eclipse E600 microscope equipped with a Nikon digital camera. A ruler was also photographed at the same magnification for determination of body length.

### Brood size assay

After one generation's treatment by *W02B12.9* RNAi or control RNAi, five to seven L4 staged F1 hermaphrodites were placed onto corresponding RNAi plates. The same animals were transferred to fresh plate every 24 hours until reproduction ceased. The number of eggs and the number of hatched worms were counted everyday after L4 stage for brood size determination.

### Microscopy and imaging

Images were captured by a Nikon digital camera attached to a Nikon Eclipse E600 microscope. Images used for body length measurement were directly captured from worms on the plates. For observation at higher magnification, animals were mounted onto a piece of 2% agar and anaesthetized with 1% levamisole in M9 solution, then were examined under the microscope and photographed [39].

### Life span assay

Life span was defined as time period between L4 stage and the day worm was dead [40]. After three or four days' feeding on bacteria containing *W02B12.9* RNAi or l4440 RNAi vectors, more than sixty L4 staged hermaphrodites of F1 generation were transferred onto fresh prepared RNAi plate respectively. The same animals were transferred everyday onto fresh plates during reproductive period to avoid interference of offspring, and every two or three days afterward until none were alive. Worms that do not respond to gentle nose touch with a platinum wire were considered dead. Worms that crawled off the plate or died from hatched embryos in the uterus were scored as censored data. The Graphpad Prism software was used to compare survival curves for statistical analysis.

### Paraquat sensitivity test

After one generation's RNAi treatment, around thirty three-day old F1 adults were put into 96-well plates with M9 solution containing 100 mM paraquat (1, 1′-dimethyl-4,4′-bipyridinium dichloride, Sigma-Aldrich). Dead worms were counted every 5 hours up to the 25-hour time point. Worms that do not respond to gentle nose touch with a platinum wire were considered dead.

### Quantitative real-time PCR

After one generation's RNAi treatment, ten L4 staged F1 hermaphrodites were transferred onto corresponding RNAi plates. Four days later, mix-staged worms on the plate were washed into a 1.5 ml tube with ddH_2_O and were centrifuged twice at low speed (200 rpm) to remove bacteria. Total RNA from about 10 to 15 µl worm pellet was isolated using trizol reagent (Invitrogen). Intact RNA was checked by running a 2% agrose electrophoresis and was quantified spectrometrically (Beckman Coulter DU-800). Obtained RNA was converted to cDNA using the PrimeScript RT reagent kit (Takara). 1 µl of the resulting cDNA was used for quantitative real-time PCR using the SYBR Premix EX Taq™ (Takara) and the ABI PRISM 7700HT Real-Time PCR System (Applied Biosystems). The qRT-PCR conditions were: 95°C for 30 s, followed by a 40-cycles of 5 s at 95°C and 30 s at 60°C. Specificity of the amplified product was ensured by melting curve analysis. *W02B12.9* mRNA levels in worms are normalized to the internal control *act-1*. Premiers for *act-1* are:

Forward primer: 5′-CCGCTCTTGCCCCATCAAC-3′;
Reverse primer: 5′-AAGCACTTGCGGTGAACGATG-3′.

Primers for *W02B12.9* are:

Forward primer: 5′-GGCGAATTTTCTTGCAGGCT-3′;
Reverse primer: 5′-TCAACTCGAATGACCTCCTT-3′.

Primers were designed to span across one intron to make sure that products can only be amplified from cDNA, not genomic DNA, in order to avoid interference of the latter. The qRT-PCR experiments were repeated three times using independent RNA preparations.

### Statistical analysis

Statistical analyses for body length, brood size, locomotive rate and qRT-PCR were performed by Student's *t* test. Life span and paraquat sensitivity statistics were analyzed using Kaplan-Meier survival method followed by log-rank test. The Graphpad prism 5 software was used for analysis.

### Internet resources and software

Gene and protein sequences were obtained and submitted to blast searches at ncbi (http://www.ncbi.nlm.nih.gov/) and worm base (www.wormbase.org). Primers were designed using the Primer 5 software and sequence alignments were created using BioEdit v 7.0.9. Predicted transmembrane regions of proteins were obtained from Uniprot database (http://www.uniprot.org/).

## Results

### *C. elegans W02B12.9* is the only homologue of vertebrate *mitoferrin 1/2* and yeast *MRS3/4*

**Figure 1:**
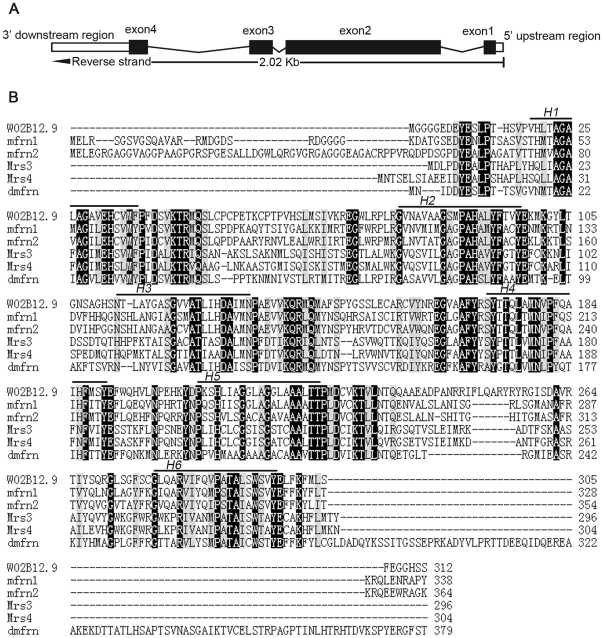
Predicted *W02B12.9* gene structure and the amino acid sequences alignment of mitoferrin proteins from species. A) Predicted gene structure of *W02B12.9*. Black box, exon; white box, untranslated region; line between boxes, intron. B) Alignments of the amino acid sequence of W02B12.9 with human mitoferrin 1 (mfrn1) and mitoferrin 2 (mfrn2), yeast Mrs3 and Mrs4, and *Drosophila* dmfrn. Identity (*black boxes*) and similarity (*grey boxes*) in the six sequences are highlighted and bars above the sequences indicate predicted transmembrane helices H1–H6. GeneBank accession numbers: W02B12.9 NP_496447.1; mitoferrin 1 NP_057696.2; mitoferrin 2 NP_112489.3; Mrs3 NP_012402.1; Mrs4 NP_012978.1; dmfrn NP_651600.1.

Invertebrates were reported to have one *mitoferrin* [24], [25]. We performed blast searches against Worm protein database using mitoferrin proteins of *Saccharomyces cerevisiae*, *Drosophila melanogaster* and *homo sapiens.* One hypothetical protein called W02B12.9 was revealed, which shows similarity to members of mitochondrial solute carrier family. It localizes to the reverse strand of chromosome 2, has four exons, and is predicted to have 321 amino acids and a molecular weight of 34.1 kD (Figure 1A). The amino acid sequence identities between W02B12.9 and its counterparts of human, drosophila or yeast species are more than 40% (Figure 1B). The predicted transmembrane regions of mitoferrin from these species were obtained and the alignment of these regions also suggests that they are conserved in evolution (Figure 1B). *W02B12.9* should be worm *mitoferrin* and there are several evidences: Firstly, mitoferrin proteins are physiologically important. As multicellular organism worms should have its mitoferrin and W02B12.9 is the most likely candidate because of its high amino acid sequence identity to mitoferrin from other species. Secondly, the existence of *W02B12.9* mRNA was confirmed (wormbase), and was also shown by our reverse transcription PCR experiments. Thirdly, the transcriptional green fluorescent protein (GFP) fusion reporter suggested that W02B12.9 was expressed from embryo to adult in many tissues including intestine and neurons [41]. Finally, proteins involved in iron homostasis regulation and iron-sulfur cluster biogenesis are usually conserved in evolution [1]. Based on these evidences, it is very likely that *W02B12.9* is the worm *mitoferrin* and we call it *mfn-1* for convenience according to the nomenclature of *C. elegans.* (http://www.cbs.umn.edu/CGC/nomenclature/).

### RNAi treatment of *mfn-1* leads to slow locomotion and small body size

**Figure 2:**
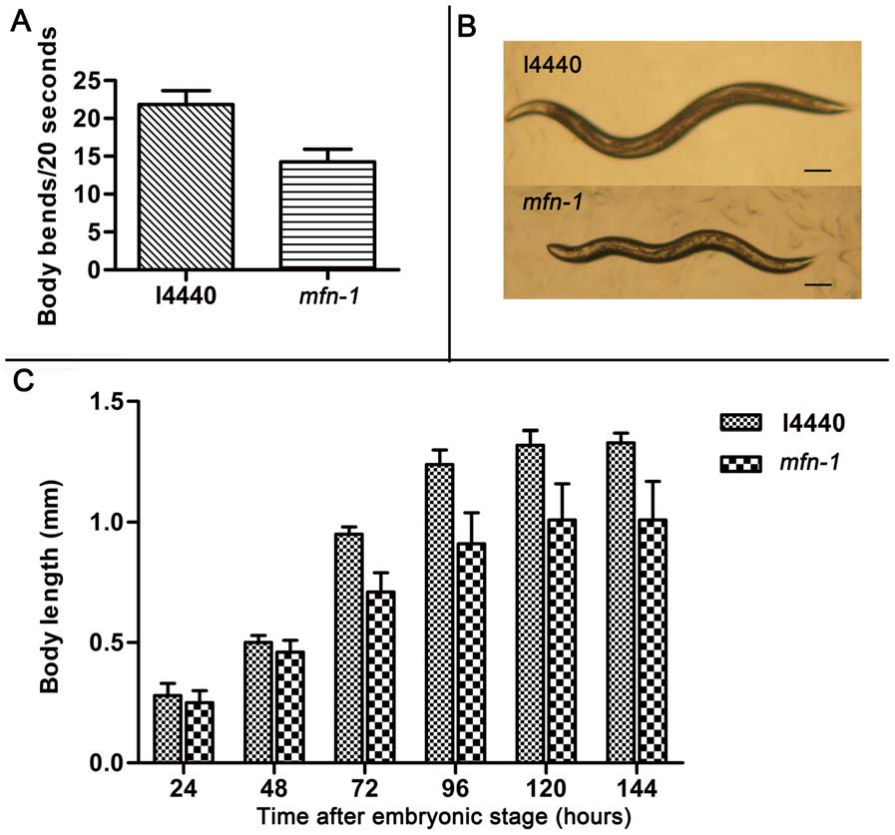
Mfn-1 RNAi treated worms have decreased locomotive activity and small body size. A) Average body bends per 20 s of *mfn-1* RNAi individuals are 14±1, and that of l4440 RNAi animals are 22±1, p<0.0001. The histogram represents average ± SD of three independent experiments with five animals for each measurement. B) Representative photographs of 2-day old adults feeding on l4440 RNAi and *mfn-1* RNAi vectors. Scale bars = 100 µm. C) Measurement of body length every 24 hours during growth. The final body length is 1.01±0.16 mm of *mfn-1* RNAi worms, and that of control is 1.33±0.04 mm, p<0.0001. The histogram represents average ± SD of five independent experiments with more than 30 animals measured respectively each day.

RNAi technology is frequently used in *C. elegans* for rapid study of gene functions in vivo. It can be performed by feeding worms with bacteria expressing dsRNA, by injecting dsRNA into gonad, by soaking worms in dsRNA solution or by making transgene animals expressing dsRNA in vivo. Of these methods, feeding RNAi is the most common used because of its simplicity [38]. We found that worms feeding on E.coli HT115(DE3) strain containing the *mfn-1* RNAi construct had slowed movements. We transferred five to seven one-day old hermaphrodites onto NGM plates without food (worms often move regularly in the absence of food) and counted their body bends in 20 seconds. The results showed that body bends rate was decreased by about 35% by RNA interference of *mfn-1* [Figure 2A]. However, the locomotive activity was maintained for a longer time, which declined slowly and finally overshadowed that of control at old age (our observations). *Mfn-1* RNAi worms had small but well proportioned body size, and appear thin and pale (Figure 2B). Body length was measured every 24 hours during worms' growth and the results showed that growth rate was decreased by *mfn-1* RNAi treatment, but the growth period was neither prolonged nor shortened (Figure 2C).

Small body size was often associated with increased longevity. For example, worm strain with mutation in *eat-2* gene, which mimics dietary restriction (DR), have small body size as well as increased lifespan [42]. And mitochondrial dysfunctions caused by mutations in genes such as *clk-1*, *isp-1*, and *sod-2*, lead to increased longevity and slowed growth [43], [44].

However, small body size was not prerequisite for lifespan extension, and it can be uncoupled from longevity some times as was reported [45]. Further studies are needed to reveal whether there are any correlations between lifespan and body size in *mfn-1* RNAi worms.

### *Mfn-1* RNAi results in reduced brood size and abnormal gonad morphology

**Figure 3:**
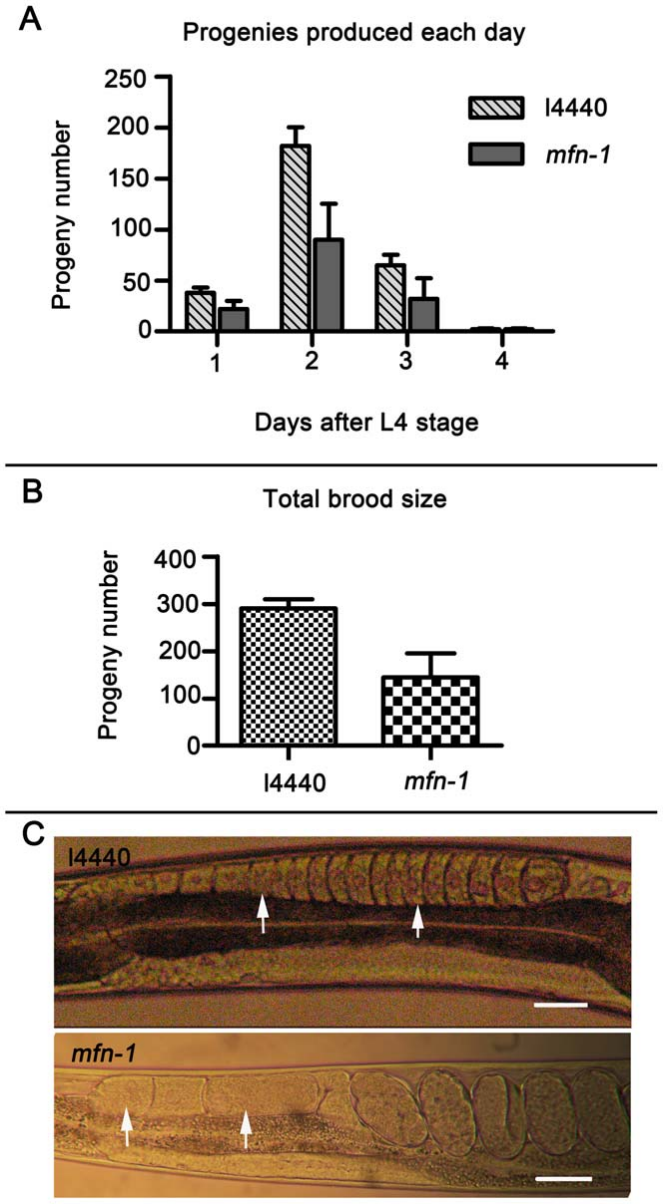
*Mfn-1* RNAi results in reduced brood size and abnormal gonad morphology. A) Progeny number was measured every day after L4 stage. The results represent five independent experiments with totally 30 worms measured respectively each day. The histogram represents average ± SD. B) Total brood size measured. Average brood size of l4440 RNAi worms is 290±20, while the brood size of *mfn-1* RNAi worms is 144±51, p<0.0001. Thirty brood sizes were measures respectively. C) Gonad images of 2-day old adults. Arrow indicates oocyte, scale bar = 30 µm.

It was reported that mitoferrin is essential for male fertility of *Drosophila melanogaster* [25]. We also found that *mfn-1* RNAi worms produced fewer progenies each day than control (Figure 3A), and the total progenies produced were significantly decreased too (Figure 3B). In order to investigate the cause of the reduced brood size, we examined age matched adults under microscope. Images were obtained using a Nikon Eclipse E600 microscope equipped with a Nikon digital camera. Two-day old *mfn-1* RNAi treated hermaphrodites were found to have fewer matured oocytes than control at the proximal side of the gonad arm (Figure 3C). In l4440 RNAi worms the oocytes are tightly arranged and exhibited regular structures, while in *mfn-1* RNAi worms they are loosely arranged and often showed abnormal structures (Figure 3C). Although brood size was affected by RNA interference of *mfn-1*, the reproductive period was not influenced. We found that worms began or stopped laying eggs on the same day as did control. Previous studies showed that germ line deficiency can result in extended lifespan [46]. It is therefore interesting to investigate in future studies whether germ line plays some roles or not in lifespan modulation of *mfn-1* RNAi worms.

The number of mitochondria increased six fold during transition from L4 to adult, perhaps due to increased demand for energy during germ line development [47]. And during the reproductive period, worms lay hundreds of eggs in the first two days (Figure 3A). Therefore, the amount of iron may be insufficient to meet the needs of germ line development and offspring production, which may contribute to the reduced brood size of *mfn-1* RNAi worms.

### *Mfn-1* RNAi worms exhibit significantly extended lifespan and increased sensitivity to paraquat

**Figure 4:**
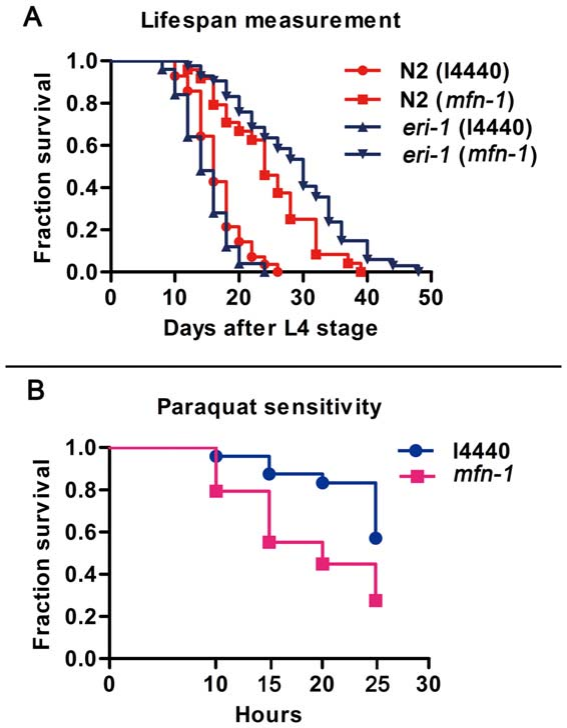
*Mfn-1* RNAi worms show extended lifespan despite their hypersensitivity to paraquat solution. A) Lifespan measurement of worms. Lifespan was measured as described in Materials and Methods. Proportion of surviving animals is plotted against days of adult life. Average lifespan of N2 wild type worms treated by *mfn-1* and control RNAi are 24.5 and 16.6 days respectively, and that of *eri-1* strain are 29.1 and 14.1 days respectively. The date indicates that lifespan is significantly increased by RNA interference of *mfn-1* in both N2 and *eri-1* strains, p<0.0001. B) Paraquat sensitivity assay. Three-day old adults were used for the test and paraquat concentration was 100 mM. *Mfn-1* RNAi worms are more sensitive to paraquat mediated oxidative stress. P<0.0001, P value represents log rank test for the experiment, comparing survival time of the l4440 RNAi and *mfn-1* RNAi worms. The figure shows results of one representative experiment. More results are shown in Figure S2.

Gene mutations affecting mitochondria function can sometimes increase longevity in *C. elegans* as reported [47]–[49]. In accordance with previously studies, we found that lifespan was significantly extended by down-regulation of mfn-1, a predicted mitochondrial inner membrane protein (Figure 4A). We performed the tests many times and observed an increase in longevity by 50% to 80% in N2 strain, the representative results were shown in Table 1 and Figure S1.

**Table 1:** Summary of the results of lifespan assays.

Experiment Number	RNAi Treatment	Maximum Lifespan	Median Lifespan	Mean Lifespan	P	N
1	l4440	26	16	16.6		60
	*mfn-1*	39	24	24.5	<0.0001	60
2	l4440	26	16	17.0		55
	*mfn-1*	46	28	27.4	<0.0001	55
3	l4440	27	16	16.2		60
	*mfn-1*	42	27	26.6	<0.0001	60

Data are combined from three independent experiments and lifespan was measured at 20°C. P values were obtained from Log-rank tests.

Although RNAi technology is an important reverse genetic approach to the study of gene functions in *C. elegans*, it could not completely inhibit gene expression and the inter-experiments variations are often large [49], [50]. For example, *daf-2* mutant doubles lifespan while *daf-2* RNAi only increased lifespan by less than 30% as reported [49]. Besides, some tissues like neurons are refractory to RNAi. Nevertheless, we observed consistent and robust lifespan extension in our experiments. In further studies using the RNAi hypersensitive strain *eri-2*, whose neurons are also sensitive to RNAi, more than doubled lifespan was uncovered by *mfn-1* RNAi treatment (Figure 4A and Table 1). Therefore, down-regulation of *mfn-1*, at least to some extent, can extend lifespan significantly in *C. elegans*.

In order to investigate whether the extended life span was resulted from increased protection from oxidative stress or not, we studied how *mfn-1* RNAi affects survival by treating worms with paraquat, which produces superoxide radicals through a radical ion intermediate and was widely used to assess sensitivity to oxidative stress. We monitored worms' survival in paraquat solution every five hours and found that *mfn-1* RNAi worms were more sensitive to paraquat at multiple time points throughout the experiments (Figure 4B). More detailed results were shown in Figure S2. Therefore, animals have decreased protection from oxidative stress, at least shown by this paraquat sensitivity test. This is somewhat inconsistent with the oxidative stress theory of aging, which states that organisms age because cells accumulate free radical damages over time. However, this theory is confronting with growing challenges [51]–[55]. For example, worm strains with mutations in sod genes have extended lifespan and show increased sensitivity to oxidative stress [44]. Low concentration of paraquat or mild heat shock treatment can extend lifespan and at the same time increase ROS levels in *C. elegans* [56]–[58]. Furthermore, adding antioxidants such as N-acetyl-cysteine or vitamin C can block lifespan extension in some long-lived mutants [56].

Further study is needed to reveal the pathways or factors responsible for these phenotypes, which may contribute to our understanding of aging and perhaps have implications in treatment of diseases with mitochondrial iron accumulation.

**Figure 5:**
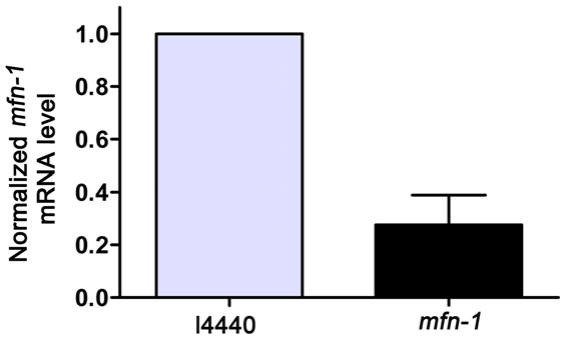
Reduction of *mfn-1* mRNA levels is confirmed by qRT-PCR. The decrease of mRNA level after RNAi treatment is assessed by qRT-PCR. The data shows that *mfn-1* mRNA level in animals treated by *mfn-1* RNAi is less than 30% the level of control worms, p<0.0001. Histogram represents normalized mRNA levels and error bar indicates standard deviation of three independent experiments.

Finally, reduction of *mfn-1* mRNA levels by RNA interference was confirmed by qRT-PCR (Figure 5). To further address the issue that these results might be caused by off-targeting effects, another RNAi vector targeting a different region of *mfn-1* was constructed. Similar phenotypes such as small brood size, small body size and increased longevity were observed by RNAi treatment using this vector, thus the off-targeting effects was excluded (Figure S3).

## Discussion

Mitochondrial iron homeostasis is essential for cell functions, such as heme and iron-sulfur cluster biogenesis. Little was known about the mechanisms regulating mitochondrial iron homostasis. Iron transport into mitochondria is facilitated by mitoferrin 1/2 which belong to the mitochondrial solute carrier family. As the gate controlling iron input, mitoferrin 1/2 must play important roles in iron regulation. Using *C. elegans* as model, we found that animals displayed phenotypes of small body size, reduced fecundity, slow locomotion and increased sensitivity to paraquat by *mfn-1* RNAi treatment. Surprisingly, lifespan was significantly extended. These results indicate that mitoferrin is essential for modulation of growth, reproduction, locomotion and longevity. Some of the phenotypes we observed such as reduced brood size, slow growth and embryonic lethality have been reported by some large-scale genome RNAi screens [50], [59], [60].

There are also several large-scale genome RNAi screens for longevity genes in *C. elegans* [47], [49], [61]. More than one hundred genes are uncovered that can increase lifespan by 10% to 90% when down-regulated. These genes are involved in a diversity of biochemical processes such as protein turnover, reproduction, energy metabolism, signaling, gene expression, and molecular transport. Those screens cover more than 80% of all 19000 *C. elegans* open reading frames at the most. Thus, not all genes are screened for longevity.

The efficiency of RNAi in suppressing gene expression could be variable between experiments and it is also possible that if two genes have identical or near identical sequences, both of them could be down-regulated by RNAi. Besides, cross-contamination also exists sometimes. There are false-positive and false-negative results in these screens. Particularly, false-negative rates are usually more than 50%, and in the study of postembryonic phenotypes the rate can surpass 80% as reported [50]. Some well known longevity genes were not revealed by these screens. Because of these reasons, it is not a surprise that *W02B12.9* (*mfn-1*) was not revealed by these longevity screens. To further confirm the lifespan extension observed in *mfn-1* RNAi worms, we used the RNAi hypersensitive strain *eri-1*, of which neurons are also sensitive to RNAi. The result showed that lifespan was more than doubled in this strain by *mfn-1* RNAi treatment. To address the possibility that the result might be caused by off-targeting effects, we constructed another vector targeting a different region of *mfn-1* and similar phenotypes including lifespan extension were observed by using the new vector. Therefore, the result was not caused by off-targeting effects.

The phenotypes such as small body size, reduced fecundity, and slowed locomotion were found to be associated with many longevity mutants, such as *clk-1*, *nuo-2*, and *isp-1* which affect mitochondria function, and with *eat-2* mutant that mimics dietary restriction.

Although *mfn-1* RNAi worms were smaller and produced fewer progenies than control, their growth and reproduction periods seem normal, neither prolonged nor shortened. It was reported that the decreased body size and brood size can be uncoupled from longevity in some cases and are not prerequisite for lifespan extension [62]. Further studies are needed to reveal whether there are any correlations between lifespan and body size or brood size in *mfn-1* RNAi worms.

Previous studies showed that mitochondria dysfunction could activate some unknown compensation pathways and led to increased lifespan as a result [63]. This might be one of the reasons why lifespan was extended in *mfn-1* RNAi worms. However, different mutants that cause mitochondrial dysfunction may activate different compensation pathways. For instance, some longevity mutants depend on daf-16 to increase lifespan, while most others don't [48]. Furthermore, some mutants affecting mitochondria function lead to decreased lifespan, such as *mev-1* and *gas-1* [63]. This suggests the complexity of mitochondria dysfunction mediated pathways in the modulation of lifespan.

During the development and reproductive period worm grows rapidly and lays hundreds of eggs per day. A large amount of iron may be needed to maintain these biochemical processes, and the quantity may be insufficient if mfn-1 protein was down-regulated by RNAi. This might explain why *mfn-1* RNAi worms have phenotypes such as small body size, reduced fecundity, and slowed locomotion. When the rapid growth and reproduction ceased, less iron should be sufficient to maintain normal functions. If the amount of iron imported exceeds the ability of mitochondria can use, it may deposit in mitochondria and interfere with normal functions. This hypothesis was supported by the fact that iron was accumulated in tissues of aged animals. Therefore, *mfn-1* RNAi treatment might contribute to lifespan extension by preventing aging associated mitochondrial iron accumulation.

Iron homeostasis disruption is associated with many genetic diseases, such as Fiedreich's ataxia, Alzheimer's Disease (AD), Parkinson's disease (PD) and Multiple Sclerosis (MS). It was reported that mitoferrin was up-regulated in yeast and mouse models of Fiedreich's ataxia [29], which suggests its involvement in the pathogenesis of diseases with mitochondrial iron accumulation. Down-regulating the expression of mitoferrin or partially blocking the iron import channel by drugs or other approaches in the future should alleviate the symptoms of patients by preventing iron accumulation. In addition, iron chelators are being used for treatment of mitochondrial iron accumulation related diseases and are demonstrated can alleviate patients' symptoms [64]–[66].

In the studies we found that *mfn-1* RNAi worms were more sensitive to paraquat, indicating decreased protection from oxidative stress. This is somewhat inconsistent with the oxidative stress theory of aging, which states that organisms age because cells accumulate free radical damages over time. However, this theory is confronting with growing challenges which suggests the complicated relationship between ROS and aging.

We believe that further investigation into the pathways or factors responsible for the extended lifespan and other phenotypes should contribute to our understanding of aging and may also have implications in the treatment of mitochondrial iron disorder related diseases.

## Supporting Information

Figure S1 **Lifespan of** ***mfn-1*** **RNAi and l4440 RNAi treated worms.** A, B and C represent three independent experiments and the results show that lifespan was markedly extended by *mfn-1* RNAi treatment at 20°C. Lifespan was measured as described in Materials and Methods. Proportion of surviving animals is plotted against days of adult life. P<0.0001 in all the three measurements, values of P are obtained from log rank test. The data from all the three independent experiments are summarized in Table 1.
(TIF)

Figure S2 **Paraquat sensitivity tests of** ***mfn-1*** **RNAi and l4440 RNAi worms.** A, B and C represent three independent experiments. Three-day old worms were used for the test and paraquat concentration was 100 mM. P<0.001, P values represent log rank test for each of the experiments, comparing survival time of the l4440 RNAi and *mfn-1* RNAi animals.
(TIF)

Figure S3 **Phenotypes of worms feeding on RNAi vector targeting a different region of** ***mfn-1*** **.** A) Body size was decreased by *mfn-1* RNAi treatment using this vector. Average body length is 1.03±0.04 mm of *mfn-1* RNAi worms, and that of control is 1.30±0.01 mm, p<0.0001. The histogram represents average ± SD of three independent experiments with 30 animals measured respectively. B) Total brood size measured. Average brood size of l4440 RNAi worms was 293±13, and the brood size of *mfn-1* RNAi worms was 132±36, p<0.0001. Ten brood sizes were measured respectively. C) Lifespan measurement of worms. Average lifespan of N2 wild type worms treated by *mfn-1* and control RNAi were 23.2 and 16.1 days respectively, p<0.0001, n = 60. The experiments were repeated three times.
(TIF)
