# Supplementary figures and images for: Reduction of Mitoferrin Results in Abnormal Development and Extended Lifespan in Caenorhabditis elegans
Source: PLoS One. 2012 Jan 11;7(1):e29666. doi: 10.1371/journal.pone.0029666 (PMC3256167; doi:10.1371/journal.pone.0029666)

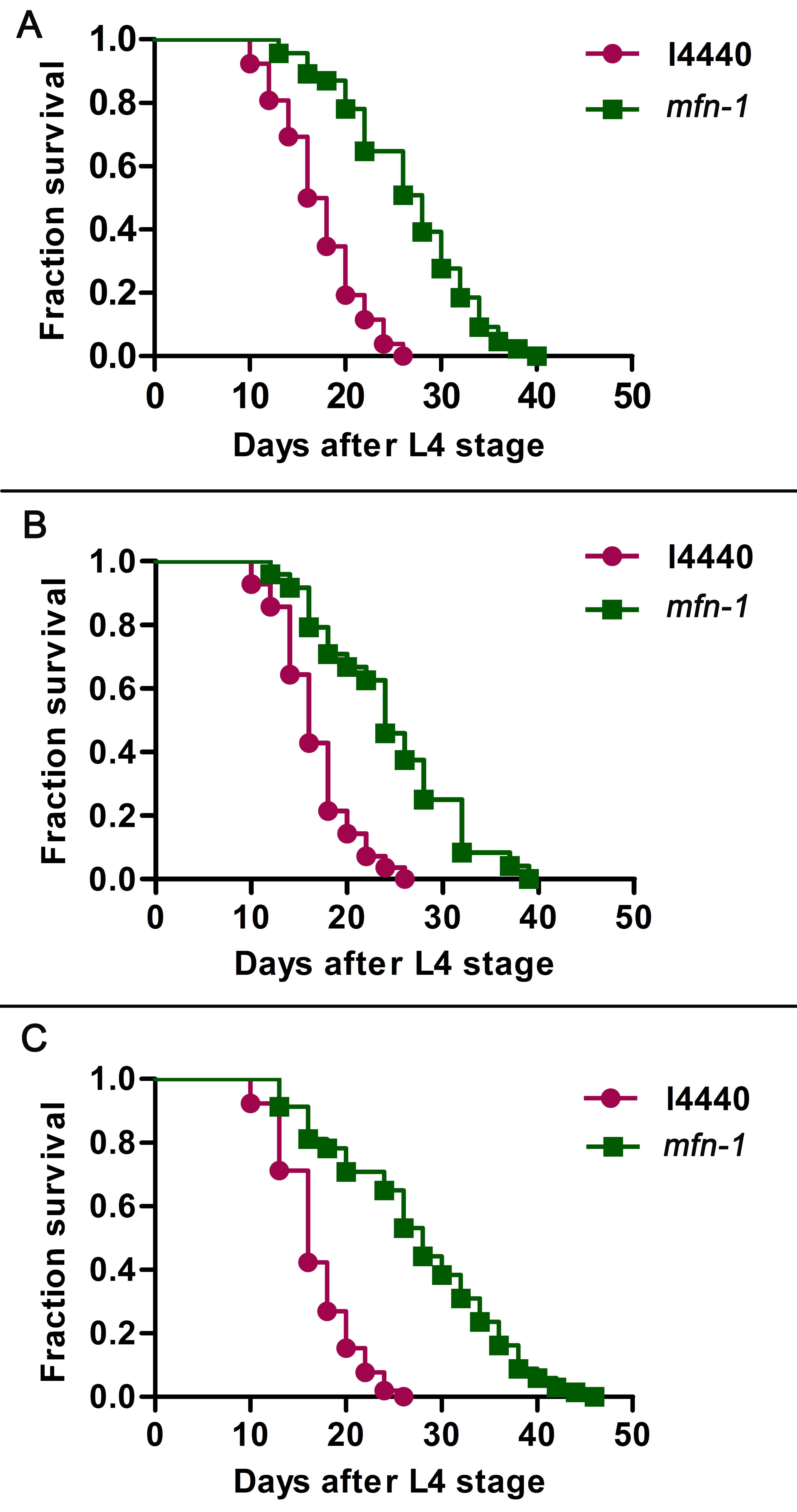

Supplement: Figure S1 — Lifespan of mfn-1 RNAi and l4440 RNAi treated worms. A, B and C represent three independent experiments and the results show that lifespan was markedly extended by mfn-1 RNAi treatment at 20°C. Lifespan was measured as described in Materials and Methods. Proportion of surviving animals is plotted against days of adult life. P<0.0001 in all the three measurements, values of P are obtained from log rank test. The data from all the three independent experiments are summarized in Table 1. (TIF) [file pone.0029666.s001.tif]

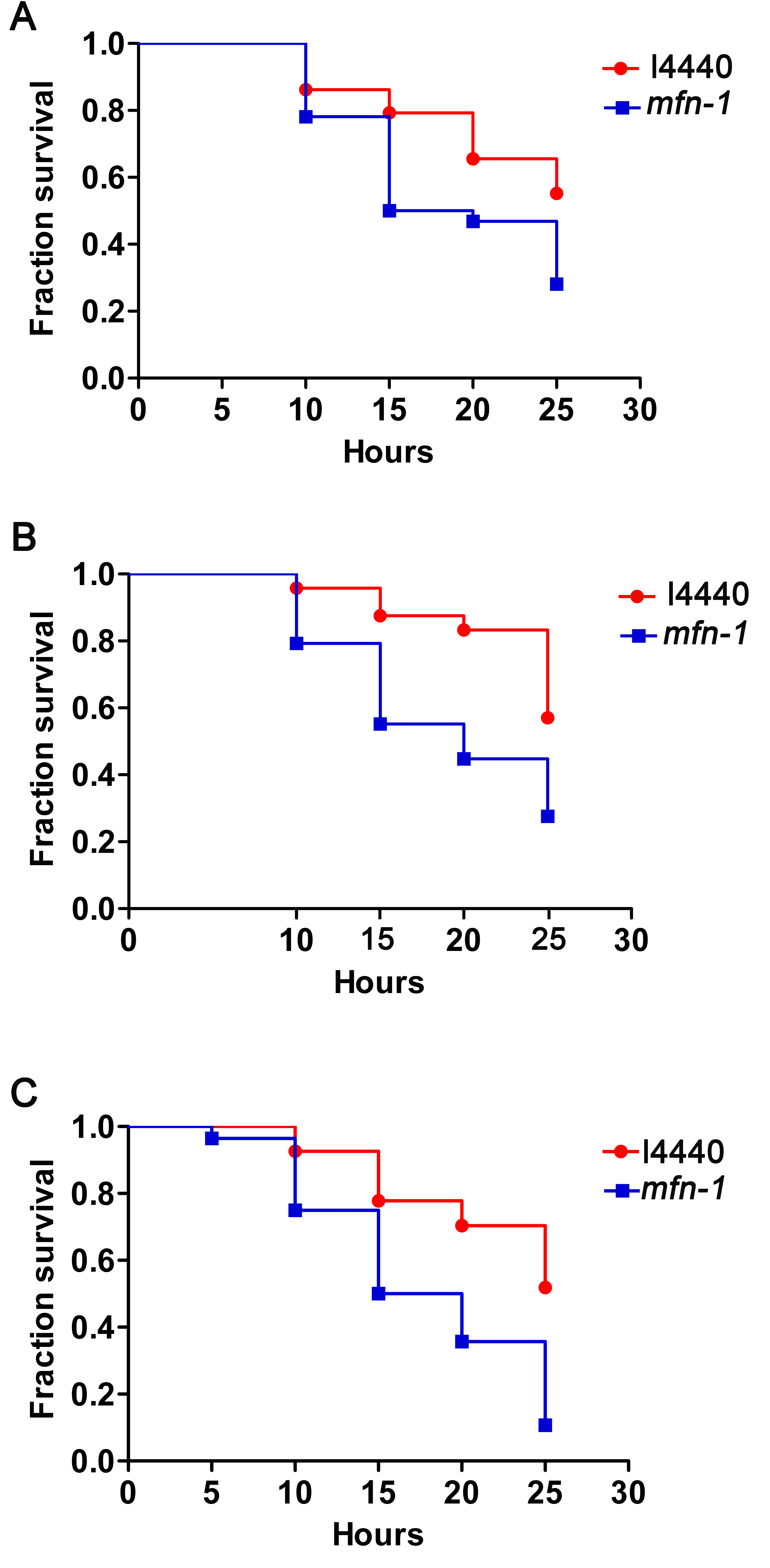

Supplement: Figure S2 — Paraquat sensitivity tests of mfn-1 RNAi and l4440 RNAi worms. A, B and C represent three independent experiments. Three-day old worms were used for the test and paraquat concentration was 100 mM. P<0.001, P values represent log rank test for each of the experiments, comparing survival time of the l4440 RNAi and mfn-1 RNAi animals. (TIF) [file pone.0029666.s002.tif]

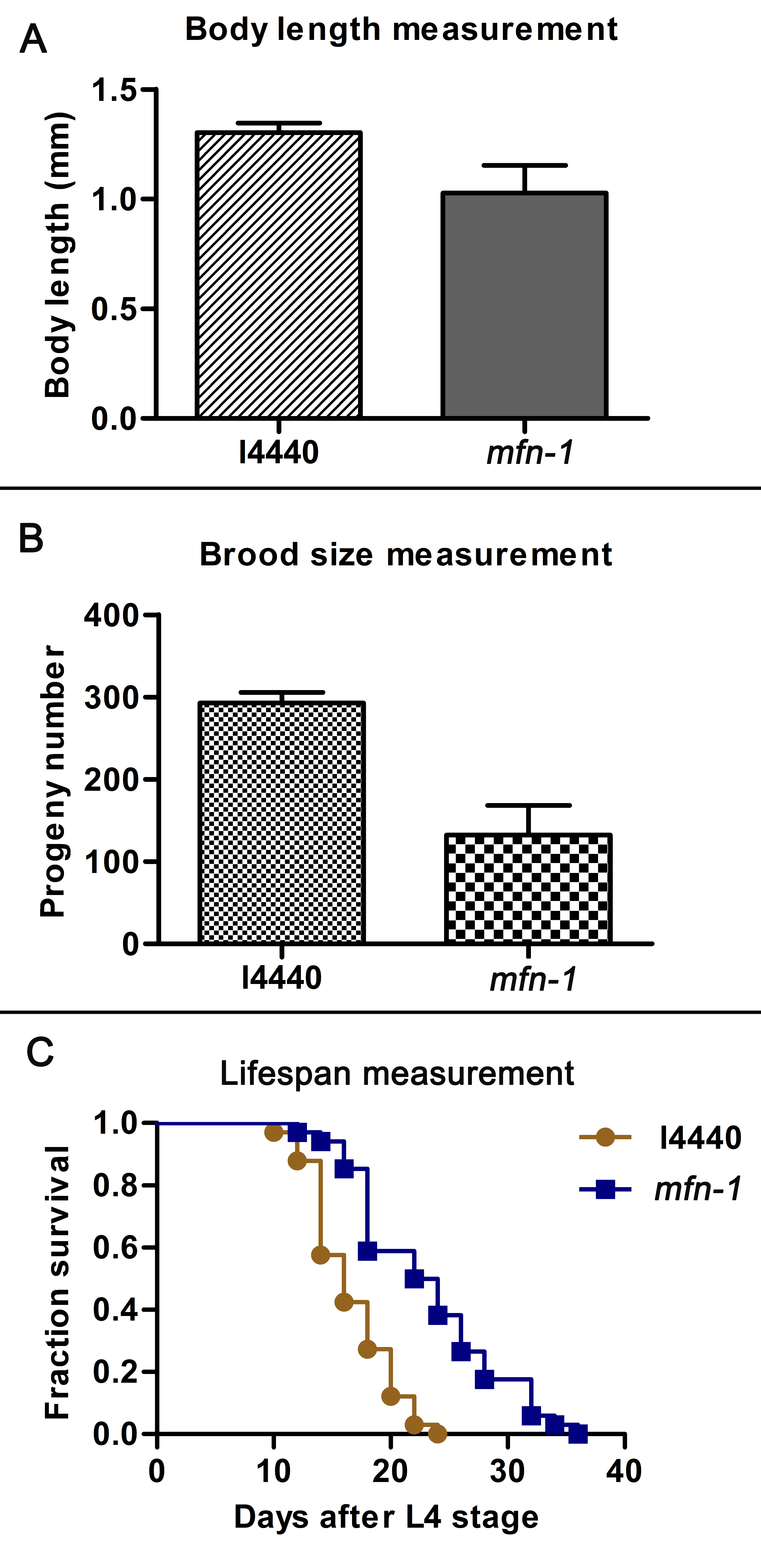

Supplement: Figure S3 — Phenotypes of worms feeding on RNAi vector targeting a different region of mfn-1 . A) Body size was decreased by mfn-1 RNAi treatment using this vector. Average body length is 1.03±0.04 mm of mfn-1 RNAi worms, and that of control is 1.30±0.01 mm, p<0.0001. The histogram represents average ± SD of three independent experiments with 30 animals measured respectively. B) Total brood size measured. Average brood size of l4440 RNAi worms was 293±13, and the brood size of mfn-1 RNAi worms was 132±36, p<0.0001. Ten brood sizes were measured respectively. C) Lifespan measurement of worms. Average lifespan of N2 wild type worms treated by mfn-1 and control RNAi were 23.2 and 16.1 days respectively, p<0.0001, n = 60. The experiments were repeated three times. (TIF) [file pone.0029666.s003.tif]
